# Deceased Donor Renal Transplantation Combined with Bilateral Nephrectomy in a Patient with Tuberous Sclerosis and Renal Failure

**DOI:** 10.1155/2019/2172163

**Published:** 2019-03-06

**Authors:** R. Novotny, J. Chlupac, T. Marada, S. Bloudickova-Rajnochova, H. Vavrinova, L. Janousek, J. Fronek

**Affiliations:** ^1^Transplant Surgery Department, Institute for Clinical and Experimental Medicine, Prague, Czech Republic; ^2^First Faculty of Medicine, Charles University, Prague, Czech Republic; ^3^Department of Nephrology, Institute for Clinical and Experimental Medicine, Prague, Czech Republic; ^4^Second Faculty of Medicine, Charles University, Prague, Czech Republic

## Abstract

**Introduction:**

A 27-year-old female patient with known tuberous sclerosis complex (TSC), polycystic kidneys with multiple large bilateral angiomyolipomas, and failing renal functions with prehemodialysis values (urea: 19 mmol/L; creatinine: 317 *μ*mol/L; CKD-EPI 0,27) was admitted to our department for pre-renal transplant evaluation. The patient was placed on the transplant waiting list as the living donor did not pass pretransplant workup and was subsequently contraindicated. Patient was placed on the “cadaverous kidney transplant waiting list”.

**Method:**

Computed tomography angiography revealed symptomatic PSA in the right kidney angiomyolipoma (AML). The patient underwent urgent transarterial embolisation of the PSA's feeding vessel in the right kidney AML. Based on the “kidney transplant waiting list” order patient underwent a bilateral nephrectomy combined with transperitoneal renal allotransplantation of a cadaverous kidney graft through midline laparotomy, appendectomy, and cholecystectomy.

**Results:**

Postoperative period was complicated by delayed graft function caused by acute tubular necrosis requiring postoperative hemodialysis. The patient was discharged on the 17th postoperative day with a good renal graft function. Patient's follow-up is currently 23 months with good graft function (urea: 9 mmol/L; creatinine: 100 *μ*mol/L).

**Conclusion:**

Renal transplantation combined with radical nephrectomy provides a definitive treatment for TSC renal manifestations.

## 1. Introduction

Tuberous sclerosis complex (TSC) is a rare neurocutaneous genetic disease with an autosomal dominant inheritance that can affect multiple organs with an approximate incidence of 1 in 5000 to 10.000 annually [1]. Two genes transmit TSC:* TSC 1* gene (chromosome locus 9q34) and* TSC 2 *gene (chromosome locus 16p13.3) [2]. In general, 50%-80% of patients with TSC present with renal pathology, angiomyolipomas (AML), renal cysts, renal carcinoma, and oncocytomas [3].* TSC 2* gene mutation is more severe than* the TSC 1* gene mutation, as it has a more severe manifestation of renal pathology [2]. Renal disease is the leading cause of mortality in adults with TSC [4]. This is due to the AMLs high incidence of haemorrhage. Furthermore, AMLs associated with TSC occur in multiples, bilaterally, grow more aggressively, and are predominantly presented in younger females when compared to isolated AML. This makes the treatment of AMLs in TSC patients much more complicated [5].

## 2. Case Presentation

A 27-year-old female patient with medical history of reoccurring hematuria led a CT angiography examination of the kidneys, revealing a polycystic kidney with angiomyolipomas. Suspicion on the TSC was made and confirmed with genetic examination revealing a TSC1 mutation in DNA in March 2012. Since then, the patient was started on a mTOR inhibitor therapy (everolimus) with dose adjustments based on blood concentrations during regular check-ups.

**Figure 1:**
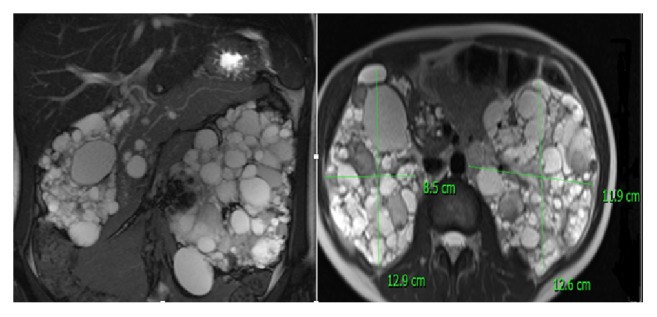
Polycystic kidneys with bilateral renal angiomyolipomas.

Patient with known TSC, polycystic kidneys with bilateral AMLs (Figure 1), failing renal functions with prehemodialysis values (urea: 18.5 mmol/L; creatinine: 317 *μ*mol/L), lung lymphangiomyomatosis (LAM), and cerebral supratentorial lesions was admitted to our department for pre-kidney-transplant evaluation in October 2012; potential living donor was patients' mother.

During 2013 patient's renal parameters showed a slight decline (urea: 18 mmol/L; creatinine: 395 *μ*mol/L). The patient was hospitalised twice for minor hematuria without the need for blood transfusion and surgical or endovascular intervention.

**Figure 2:**
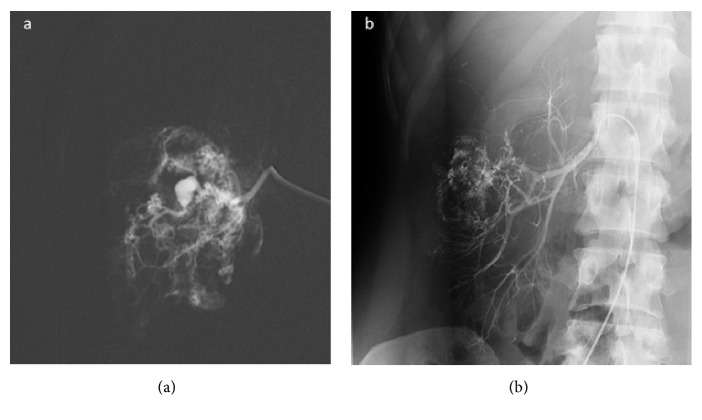
Angiography of the right kidney angiomyolipoma's (AML) pseudoaneurysm (PSA): (a) patent PSA and feeding vessel of the AML before endovascular closure; (b) occluded PSA feeding vessel of the AML.

In May 2014 patient's renal parameters declined severely (urea: 25 mmol/L; creatinine: 457 *μ*mol/L). Due to the deterioration of renal functions, renal transplantation with bilateral nephrectomy was scheduled. Before the procedure patient was put off everolimus therapy in August 2014. However, the potential living donor was contraindicated based on serology results (anti-HBs 433 IU/l). In September 2014 while still off everolimus therapy, the patient was hospitalised for massive hematuria (haemoglobin: 79 g/L) with the need for blood transfusion. Computed tomography angiography revealed symptomatic pseudoaneurysm (PSA) in the right kidney AML. The patient underwent an urgent transarterial embolisation of the PSA feeding vessel in the right kidney's AML (Figure 2). The procedure was successful. After the procedure, the patient had neither hematuria nor the need for further blood transfusion. Since then, the patient did not have any major hematuria requiring hospitalisation.

Due to the decrease of the renal parameters a native radiocephalic arteriovenous fistula was created for hemodialysis. The patient was put on the “kidney transplant waiting list”. The Czech Republic allocation system does not allow for a priority based on the high risk of bleeding.

In July 2016 the patient was admitted to our center for a cadaverous kidney transplant based on “kidney transplant waiting list” order.

**Figure 3:**
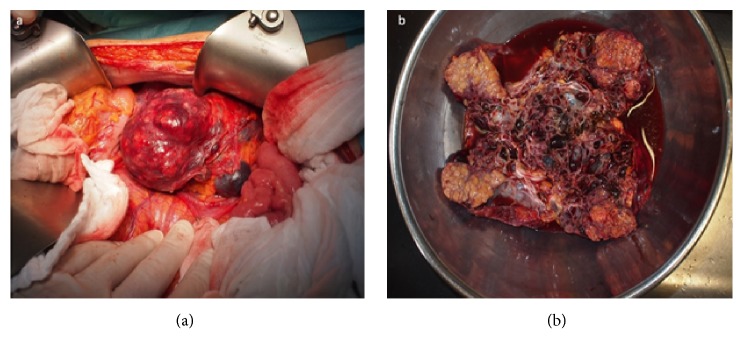
Periprocedural finding. (a) Embolised right kidney angiomyolipoma. (b) Explanted polycystic kidney.

The patient underwent a bilateral nephrectomy combined with renal transperitoneal allotransplantation of the cadaverous kidney graft with a prophylactic appendectomy, and cholecystectomy through midline laparotomy (end-to-side anastomosis of the renal graft's vein- external iliac vein, renal graft's artery-external iliac artery; ureterocystoanastomosis with 24 cm 2,4 French JJ stent) (Figure 3). The procedure was performed without any complications. Postoperative period was complicated by delayed graft function (urea: 32 mmol/L; creatinine: 797 *μ*mol/L); the patient was anuretic two days after the procedure with good graft's perfusion based on Doppler ultrasonography. This might have been caused by the time in-between the kidney graft harvest and the renal transplantation (21 hours). Grafts' biopsy was indicated, revealing acute tubular necrosis. The patient underwent two hemodialysis cycles. After six days, the patients became uretic again (1980 ml of urine/24 hours); renal functions improved drastically (urea: 26 mmol/L; creatinine: 284 *μ*mol/L). Controlled biopsy showed focal regeneration of the acute tubular necrosis of the graft. The grafts function was repeatedly checked using Doppler's ultrasonography.

The patient was discharged on the 17th postoperative day with good renal graft function (urea: 15 mmol/L; creatinine: 133 *μ*mol/L). The patient was discharged from hospital on immunosuppressive therapy: extended release tacrolimus (daily dose of 17 mg), mycophenolate (720 mg dose twice a day), and prednisone (daily dose of 20 mg). mTOR inhibitors were refrained to avoid potential wound healing complications.

Patients follow-up is currently 23 months with good graft function (urea: 10 mmol/L; creatinine: 106 *μ*mol/L). Immunosuppressive therapy was adjusted to extended release of tacrolimus (daily dose of 4,25 mg), mycophenolate (440 mg dose twice a day), and prednisone (daily dose of 5 mg).

## 3. Discussion

TSC is a rare genetic disease that can affect any organ or system in the human body. It is associated with* TSC 1 *or* TSC 2* gene mutation in up to 80% of patients [2]. The renal manifestation of the disease is present in 50%-80% of patients [3]. Renal cell carcinoma is the least common kidney manifestation of TSC with prevalence identical to a healthy population; renal cysts occur in 20% of patients with TSC [6].

Renal AMLs are benign tumours with a prevalence of 0.02%-0.1% in males and 0.22%-0.29% in females with 20% of affected patients having concomitant TSC. Out of these patients, up to 66% develop multiple renal AMLs [6]. AMLs can cause renal failure in up to 60% of patients and end-stage renal disease in approximately 15% [7]. AMLs are normally asymptomatic, but they may be presented with abdominal pain, hematuria, and progressive loss of renal function due to the loss of normal renal parenchyma leading to end-stage renal disease. They are associated with micro- and macroaneurysm that predispose patients to haemorrhage. Bleeding and life threating shock are associated with AML's size > 4 cm and aneurysms size> 0,5 cm [2, 6]. Selective arterial embolisation is the most commonly used nephron-sparing intervention. It is used as a prophylaxis for high-risk AMLs with acute bleeding and/or before nephrectomy to minimise perioperative blood loss. Criteria for selective arterial embolisation are asymptomatic AML > 8 cm or symptomatic AML > 4 cm [8]. This technique had shown a very high success rate of 93 % as demonstrated by Sawada Y et al. [9].

Renal transplantation should be combined with radical nephrectomy to remove the risk of malignant transformation and tumour growth associated with immunosuppression. Dallos G et al. proved that this is a safe and effective treatment modality [10]. This began the pursuit for an optimal immunosuppression therapy for these patients. Many novel medical therapies have been identified till present day [11, 12]. However, the most promising results are in TSC patients treated with mTOR inhibitors after renal transplantations. They are showing a good graft function with a compelling improvement of extrarenal manifestations due to mTOR inhibitors effects on TSC [13].

The timing of nephrectomy in patients with TSC is crucial. The underlying objective is to perform nephrectomy combined with renal transplant as a one-stage procedure. The main reason is to avoid an increased risk of renal insufficiency and end-stage renal failure and to avoid two-stage procedure [14]. If the mTOR inhibitors as a “first-line” therapy fail to control the AML size, selective embolisation or kidney-sparing resections are acceptable “second-line” treatments for asymptomatic angiomyolipoma [13–15]. However, in patients with life-threatening retroperitoneal haemorrhage caused by ruptured aneurysms from angiolipomas, renal nephrectomy represents a lifesaving procedure despite the accompanying complications that may arise.

In rare cases where mTOR inhibitor treatment did not prevent renal bleeding episode a bilateral nephrectomy would be performed, requiring hemodialysis up to the renal transplant [14, 16].

At our center the internal protocol in patients that are undergoing bilateral nephrectomy followed by renal transplantation is to perform appendectomy and cholecystectomy even if asymptomatic. We believe that this significantly reduces postoperative morbidity. Acute cholecystitis after kidney transplantation is a serious complication. It can be very difficult to evaluate and perform diagnosis as clinical signs could be very mild compared with severity of gallbladder affliction [17].

LAM is rare progressive, cystic lung disease affecting 30%-40% of women with TSC [18]. The understanding of the mechanistic target of rapamycin (mTOR) pathway activation in the pathogenesis of LAM had greatly affected the pharmacological treatment of LAM. Several trials such as “Cincinnati Angiomyolipoma Sirolimus Trial” and “Multicenter LAM Efficacy of Sirolimus Trial” proved that LAM patients showed a major improvement in the FEV1, forced vital capacity, and residual volume following sirolimus administration [19, 20]. The safety and efficacy of sirolimus in the treatment of LAM were shown by Takeda et al. [21]. Everolimus (mTORC1 inhibitor) had been extensively tested for other manifestations such as AML. However, the administration of everolimus led to stabilisation of FVC and improvement in FEV1 and 6-min walk distance [22]. Even though randomised studies of everolimus were not conducted on patients with LAM, there is strong evidence of its similar beneficial treatment effect on LAM as sirolimus [18].

## 4. Conclusion

The preservation of renal function and the prevention of life-threatening haemorrhage are the main goals for treating patients with TSC and AMLs. Patient with large AML should be ideally transplanted from a live donor. Endovascular treatments such as intra-arterial embolisation should be performed during the wait on the transplant list in order to minimise the chance of severe or even life-threatening bleeding. This also prevents the intraoperative bleeding as AMLs easily bleed even if gently touched during surgery. Renal transplant combined with radical nephrectomy provides a definitive treatment of the renal manifestation in these patients.
